# The use of pharmacodynamic results for recommended phase II decision making in oncology clinical trials

**DOI:** 10.1007/s00280-026-04924-7

**Published:** 2026-07-06

**Authors:** A. C. Kanhailal, M. J. J. Lucassen, T. Schutte, W. Zwart, A. D. R. Huitema, N. Steeghs

**Affiliations:** 1 Department of Medical Oncology, The Netherlands Cancer Institute, Amsterdam, 1066 CX The Netherlands; 2 Oncode Institute, Utrecht, The Netherlands; 3 Division of Oncogenomics, The Netherlands Cancer Institute, Amsterdam, The Netherlands; 4 Department of Biomedical Engineering, Eindhoven University of Technology, Eindhoven, The Netherlands; 5 Department of Pharmacy & Pharmacology, The Netherlands Cancer Institute, Amsterdam, The Netherlands; 6 Princess Máxima Center for Pediatric Oncology, Utrecht, The Netherlands; 7 Department of Clinical Pharmacy, University Medical Center Utrecht, Utrecht University, Utrecht, The Netherlands; 8 Department of Medical Oncology, University Medical Center Utrecht, Utrecht University, Utrecht, The Netherlands

**Keywords:** Pharmacodynamic analysis, First in Human clinical trial, Small-molecules, Recommended Phase II Dose

## Abstract

**Introduction:**

Pharmacodynamic analyses are increasingly important to early-phase oncology drug development, however these analyses use invasive methods to collect patient material. Yet, their role in guiding recommended phase II dose (RP2D) decisions remains unclear. Therefore, this review evaluates how pharmacodynamic analyses assisted the determination of the recommended phase II dose (RP2D) in first-in-human cancer trials of small-molecule agents.

**Method:**

First-in-human dose finding clinical trials of single-agent small-molecule anticancer therapies published between 2022 and 2025 that included pharmacodynamic assessments, were investigated. Clinical trials were evaluated for correlation of pharmacodynamic results at the analyzed doses and their influence in the RP2D decision-making process.

**Results:**

In total, 535 records were identified and 34 articles, reporting on 34 separate trials, were included. A correlation was found between the pharmacodynamic results and the analyzed dose levels in 92% (*n* = 31/34). However, only half of the clinical trials (*n* = 17/34) reported their pharmacodynamic results in the RP2D decision-making process, while 41% (*n* = 14/34) did not. In most of the clinical trials that did not use their pharmacodynamic results in the RP2D decision-making process, only one pharmacodynamic analysis was performed (*n* = 9/14, 64%) and they often determined their RP2D solely based on safety and/or tolerability (*n* = 7/14, 50%). Invasive procedures were often performed without clear utilization of pharmacodynamic data, raising ethical considerations.

**Conclusion:**

These findings underscore the need for appropriate sample sizes planning and validated, fit-for-purpose pharmacodynamic assays to ensure meaningful use in dose selection, optimizing patient benefit and trial design.

**Supplementary Information:**

The online version contains supplementary material available at 10.1007/s00280-026-04924-7.

## Introduction

As oncologic drug development increasingly depends on pharmacodynamic analyses, the scientific importance of pharmacodynamic outcomes in first in human (FIH) trials is rising [1, 2]. To perform these pharmacodynamic analyses, patients are often required to undergo invasive, non-diagnostic procedures, which are becoming more common in early phase clinical trials, particularly those involving specific biomarker cohorts. The risks of such procedures depend on multiple factors, including the type of biological matrix (e.g., blood, tissue, spinal fluid) collected, and may include serious complications such as bleeding, infection, or injury to adjacent structures (e.g., pneumothorax following a lung biopsy) [3]. Patients consent to these non-diagnostic procedures in FIH clinical trials with the understanding that they will help develop treatment options for future cancer patients and provide insight into drug response and resistance mechanisms [4–6]. 

Medical ethics committees prospectively evaluate whether the potential harm or burden to participants is ethically justified by the anticipated benefits of the knowledge that is gained. A key ethical principle is that the data obtained from these procedures directly contributes to advancing the investigational compound, and be made publicly available [1, 7]. In the regulatory context, the importance of pharmacodynamic contribution to dose selection is increasingly emphasized. For example, the Food and Drug Administration (FDA), suggests that pharmacodynamic analyses may contribute to understanding dose-response relationships for dose selection in clinical trials. Therefore, in Project Optimus, guidelines have been developed to ensure sufficient evidence supports the use of biomarkers. However, the FDA points out that pharmacodynamic data from FIH trials are insufficient for definitive dose selection [8]. Similarly, the European Medicines Authority (EMA) notes in its ‘Guideline on the clinical evaluation of anticancer medicinal products’ that pharmacodynamic endpoints, where robust and relevant, may assist in the dose selection. However, they are considered supportive rather than determinative [9]. Additionally, a more recent draft guidance from the FDA and Office for Human Research Protection, highlights the requirement for clear ethical justification when invasive procedures are planned for pharmacodynamic purposes [10]. 

Despite their importance, it remains unclear how pharmacodynamic analyses are utilized and reported in FIH trials. It has been reported that pharmacodynamic analyses are sometimes not performed due to financial, logistical and/or time-related constraints, or their results are generated but remain nonetheless unpublished [11]. Also, pharmacodynamic analyses may not be completed if compounds are de-prioritized and do not advance to subsequent clinical phases, as these analyses are often conducted only after the primary objective (e.g. maximum tolerated dose, MTD) has been established. Notably, the MTD, which is traditionally used to determine the RP2D for chemotherapeutics, does not necessarily correspond to the optimal biological dose of other drug classes such as small-molecules [12, 13]. In previous studies, 22%−61% of early phase cancer trials containing invasive non-diagnostic biopsies did not report or incompletely reported pharmacodynamic analyses [11, 14, 15]. This is likely an underestimation, given that approximately 30% of phase I trials are not published at all, and a majority include a pharmacodynamic biomarker [16]. 

Targeted small-molecule drugs typically rely on pharmacodynamic analyses to provide mechanism-based evidence for their biological activity in preclinical phases. Incorporating these analyses into the design of dose-finding clinical trials, such as FIH trials, enhances understanding of biological activity, facilitates demonstration of target engagement, and supports the development of predictive or prognostic biomarkers for small-molecules [17, 18]. Therefore, pharmacodynamic analyses can assist in determining the recommended phase II dose (RP2D). Also, pharmacodynamic insights can reveal that lower doses achieve full target engagement and can therefore be selected for dose optimization. For example, many patients treated with ibrutinib at the approved dose required dose reductions due to toxicity. While, pharmacodynamic studies showed that > 95% of BTK occupancy was reached at doses far below the approved dose, with no additional pharmacodynamic benefit at higher exposures [19, 20]. Given that targeted agents remain the dominant class in drug development in the 21 st century, it is particularly important to understand how pharmacodynamic analyses inform the determination of the RP2D in FIH trials [21]. However, the extent to which these analyses are incorporated into RP2D decisions remains unclear. For example, Goulart et al. found that only 13% of clinical trial abstracts published between 1991 and 2002 reported the use of biomarkers for the selection of the RP2D, though this may be an underestimation as abstracts often provide only limited information about biomarker results and scarcity of targeted agents at that time [22]. This review evaluates to what extent pharmacodynamic analyses are used in the determination of the RP2D in recent FIH trials of small-molecule targeted agents. In addition, the types of pharmacodynamic analyses used in the determination of the RP2D, as well as the biomaterials collected to perform these pharmacodynamic analyses, were evaluated.

## Methods

### Search strategy

A literature search was performed in MEDLINE. The search strategy included standardized keywords (MeSH) and free-text words in the title and abstract. The search terms included keywords regarding oncological disease, early phase clinical trials, pharmacodynamic measurements and small-molecules. The combination of MeSH terms and text-words was used to achieve the most comprehensive results [23, 24]. A combination of thesaurus terms and title/abstract terms, based on literature, were included in a single search strategy. This search was then optimized. Articles that were indexed with the set of keywords, but did not contain the current search terms in title or abstract, were screened to discover potential new terms. New candidates were added to the basic search and evaluated until optimal recall was achieved [25]. The literature search was restricted to English and Dutch articles only and publication date between January 2022 and July 2025. This timeframe was chosen following the advice from the EMA in 2019, to implement pharmacodynamic analyses in early phase trials, and the FDA in project Optimus in 2024 [9, 26]. The language restrictions were chosen to ensure adequate review by AK and ML. The final search strategy was reviewed using the Peer Review of Electronic Search Strategies guideline [27]. The full literature search is described in Appendix I.

### Clinical trial selection

**Fig. 1:**
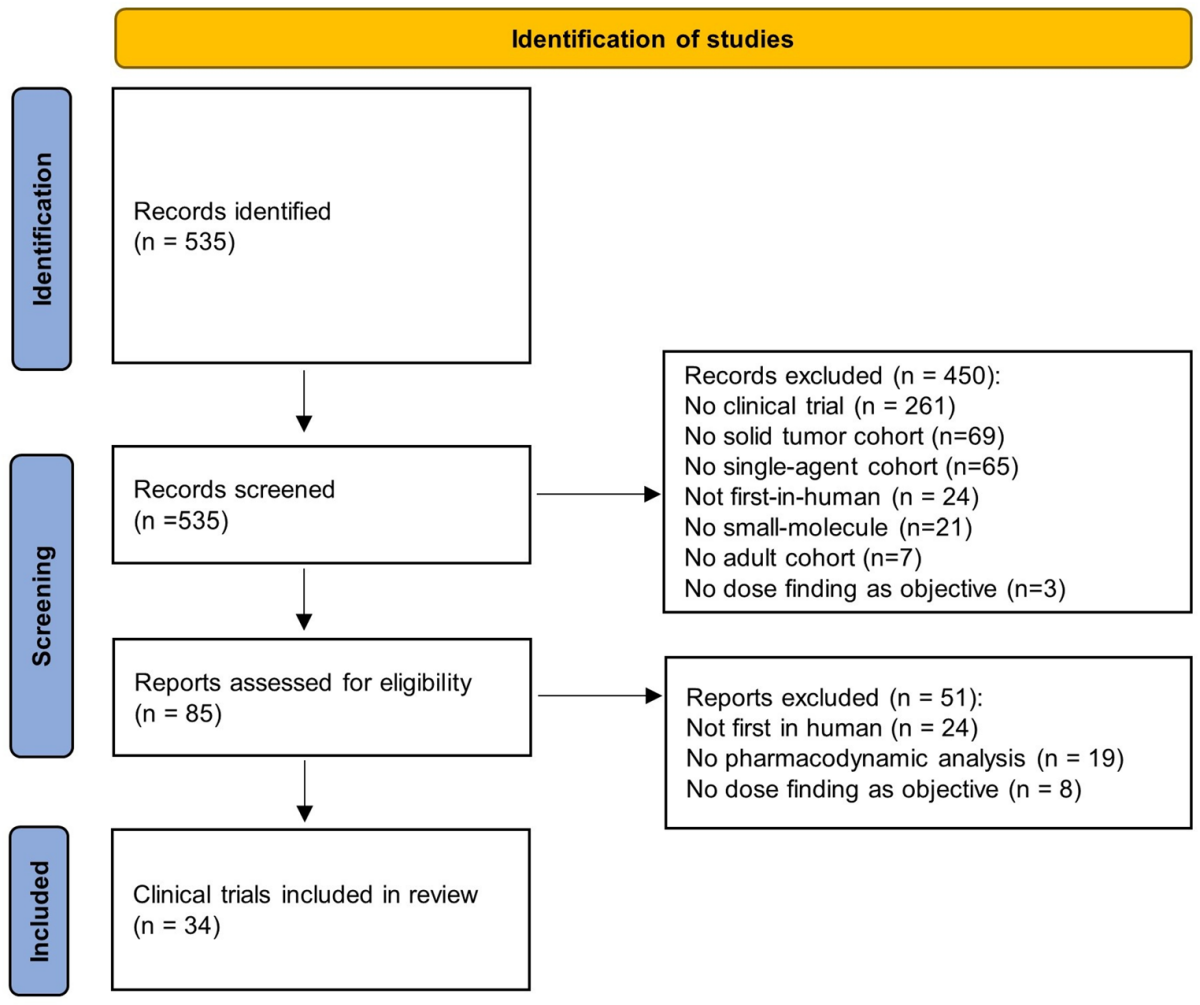
PRISMA 2020 flow diagram. Search strategy. Search has been performed on 12th August 2025. Source: Page MJ, et al. BMJ 2021;372:n71. doi: 10.1136/bmj.n71. This work is licensed under CC BY 4.0. To view a copy of this license, visit https://creativecommons.org/licenses/by/4.0/

The detailed clinical trial selection process is presented in a PRISMA flow diagram in Fig. 1. After identification of the records, duplicates were removed using EndNote 21.1 (Clarivate Analytics, Philadelphia, USA). The remaining articles were screened independently by AK and ML. Inclusion of articles was discussed among AK and ML until agreed upon. Clinical trials were included if a pharmacodynamic analysis was described in the method section of a FIH trial with a single-agent small-molecule targeted anticancer therapy for patients in solid tumors. At least one cohort with patients with solid tumors had to be included in the clinical trial, to be eligible for inclusion in this review. The term “pharmacodynamic analyses” is broadly used in clinical trials. From a pharmacological perspective, pharmacodynamics reflects drug-related biological effects. This include biomarker responses, such as target inhibition and pathway modulation, dose-response relationships, efficacy and/or toxicity. For this review, only pharmacodynamic analyses explicitly labeled by the clinical trial authors as ‘pharmacodynamic analysis’ were regarded as such. Of note: All clinical trials reported toxicity and efficacy results separately, but these endpoints were not included in this review as a pharmacodynamic analysis. Pharmacodynamic analysis was defined as the analysis of any type of molecular measurement in blood or tissue of which the outcome is a 1) direct readout of the mechanism of action (e.g. direct pathway inhibition) or 2) indirect readout of the mechanism of action (e.g. circulating tumor DNA (ctDNA), surrogate markers for pathway inhibition), and/or has an outcome correlated with 3) clinical response. Clinical trials without specification of pharmacodynamic analyses in the methods section or clinical trials without dose finding as an objective were excluded. Also, clinical trials with no explicit description of being a FIH trial, or without reference to the protocol or trial registry stating that it was a FIH trial, were excluded. Similarly, clinical trials with pediatric patients or combination therapies were excluded.

### Data extraction

The following characteristics for each included trial were extracted from each manuscript; year of publication, tumor type (e.g. all solid tumors; melanoma), drug class (e.g. estrogen receptor (ER) degrader; poly (ADP-ribose) polymerase (PARP) inhibitor), number and type of described pharmacodynamic analyses (e.g. change in biomarker expression, change in percentage target occupancy), presented results of the pharmacodynamic analysis (no results, part of the results or all results), and correlation of results (correlation or no correlation) with dose levels analyzed. The extracted data was semantically analyzed using an inductive approach, meaning that the categories were formed based on the explicit meaning of the data.

**Fig. 2:**
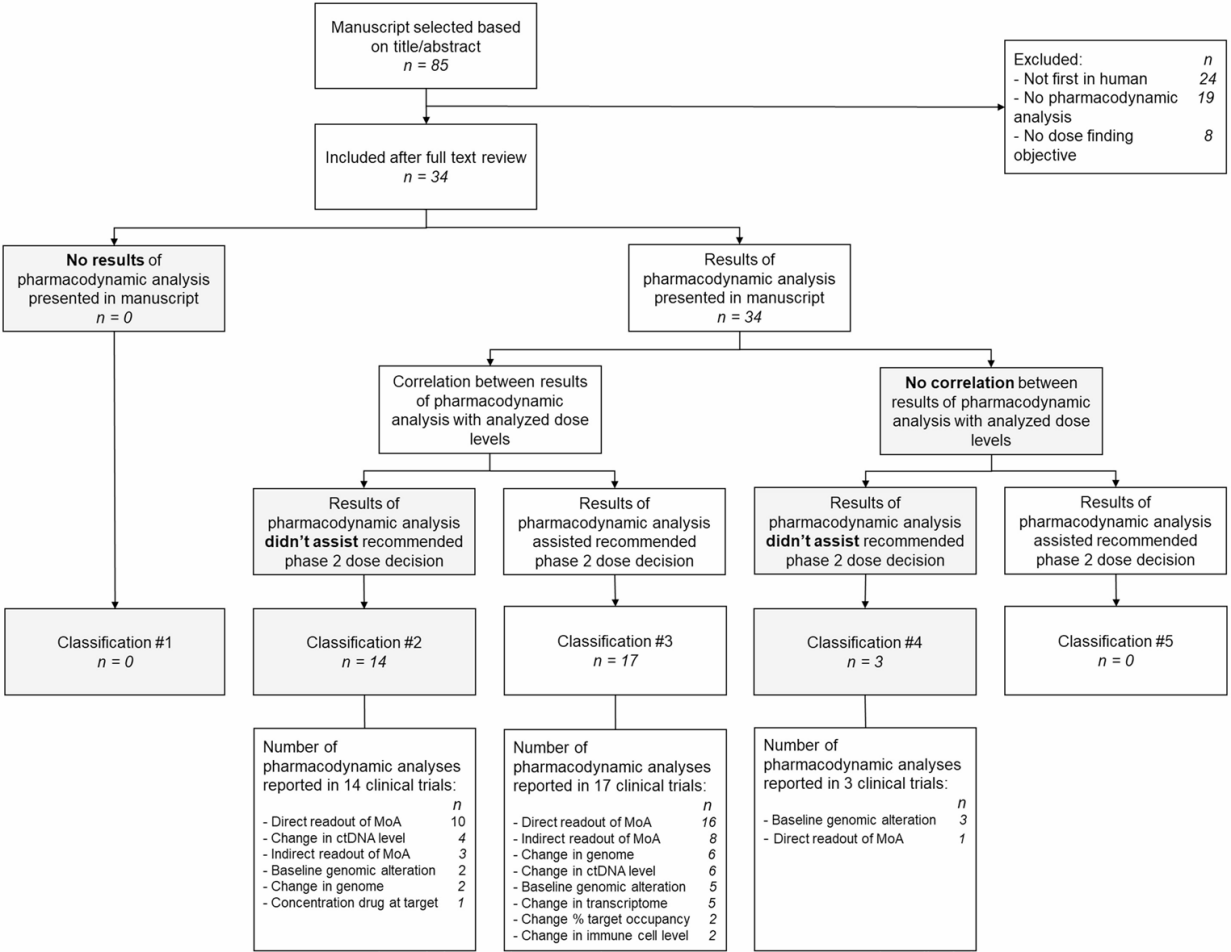
Classification flow-chart of full-text reviewed clinical trials. Clinical trials are reviewed based on use of pharmacodynamic analyses in recommended phase II dose decision making. N is number of clinical trials, unless stated otherwise. The publications were classified as follows: (1) no results presented (2), results presented and correlated with a readout of mechanism of action and/or clinical efficacy but didn’t assist in RP2D decision (3), results presented and correlated with a readout of mechanism of action and/or clinical efficacy and assisted RP2D decision (4), results presented but not correlated with a readout of mechanism of action and/or clinical efficacy and didn’t assist in RP2D decision (5), results presented and not correlated with a readout of mechanism of action and/or clinical efficacy, but assisted in the RP2D decision. % = percentage; ctDNA = circulating tumor DNA; MoA = mechanism of action

Clinical trials were classified based on the presentation of pharmacodynamic results (presented or not presented), the correlation of these results with dose levels analyzed (present or not present), and their use in the RP2D decision making (assisted or did not assist) (Fig. 2). Use of the pharmacodynamic results in the RP2D decision making was defined as any description of the specific pharmacodynamic results or ‘pharmacodynamics’ in general within the section of the manuscript that described how the RP2D was chosen. Examples are: ‘*Based on the available safety*,* efficacy*,* pharmacokinetic analysis*,* and pharmacodynamic analysis*,* 12 mg BID* (twice a day) *was identified as the RP2D and selected for dose expansion*’ [28] or ‘*The PK* (pharmacokinetics) *and PD* (pharmacodynamics) *of the 2 doses of 240 mg and 360 mg were reviewed by the Safety Review Committee (SRC) and a starting dose of 360 mg OD* (once daily) *appeared to be the most promising dose level to start treatment based on PK and PD data.*’ [29].

The publications were classified as follows: (1) no results presented (2), results presented and correlated with the analyzed dose levels, but did not assist RP2D decision (3), results presented and correlated with the analyzed dose levels, and assisted RP2D decision (4), results presented but not correlated with the analyzed dose levels, and did not assist RP2D decision (5), results presented and not correlated with the analyzed dose levels, but assisted in the RP2D decision.

## Perspectives

A total of 535 records were identified and screened for eligibility based on title and abstract. The search was performed on the 12th of August 2025. In total, 85 articles were marked as relevant and assessed based on full-text. In total, 34 (40%) articles were eligible for inclusion as they were FIH clinical trials in adult patients with solid tumors testing a single-agent small-molecule with at least one pharmacodynamic analysis described in the methods section. Of the 51 assessed but excluded articles, 37% (*n* = 19) did not describe any pharmacodynamic analyses in their methods section. However, nine of these 19 clinical trials included tumor tissue-derived and/or blood-based pharmacodynamic analyses in their corresponding protocol and/or in the clinicaltrials.gov registry [30–38]. Only one of the 19 trials was not evaluable as no Dutch or English protocol or database registry was available [39]. Underreporting of pharmacodynamic analyses using invasive non-diagnostic biopsies have been described before by Freeman et al. and Parseghian et al. [11, 15] Therefore, one might question whether these analyses were not included in the design, not performed within the trial, or if this omission was due to underreporting of pharmacodynamic results.

Trial characteristics of the included clinical trials are presented in Table 1 and Appendix II. Most clinical trials included compounds that were not first in their class (*n* = 24, 71%) and tested compounds in patients with all types of solid tumors, instead of limited to a pre-specified solid tumor type (*n* = 20, 59%). The included clinical trials described up to seven types of pharmacodynamic analyses in their methods section, however, most described only a single pharmacodynamic analysis (*n* = 15, 44%). In general, six compounds were discontinued after their FIH clinical trials (*n* = 6/34, 18%) for strategic reasons, due to observed adverse events, no signs of activity or for unknown reasons. This does not reflect the true number of phase I clinical trials of discontinued compounds as only half of oncology phase I compounds proceed to phase II trials [40, 41]. 

**Table 1:** Trial descriptives of in- and excluded clinical trials after full-text review

	No PD analysis results presented	PD analysis results presented				Total
		Correlation between results and dose levels		No correlation between results and dose levels		
		Results didn’t assist RP2D	Results assisted RP2D	Results didn’t assist RP2D	Results assisted RP2D	
	#1	#2	#3	#4	#5	
Total clinical trials included	0	14	17	3	0	34
First in class						
Yes	0	3	7	0	0	10
No	0	11	10	3	0	24
Tumor Types						
All solid tumors	0	6	12	2	0	20
One tumor type	0	7	5	0	0	12
Subspecified tumortypes	0	1	0	1	0	2
Number of pharmacodynamic analyses						
1	0	10	4	2	0	16
2	0	1	4	1	0	6
3	0	2	4	0	0	6
4	0	1	3	0	0	4
4+	0	0	2	0	0	2

Clinical trial classes were as follows: (1) no results presented (2), results presented and correlated with a readout of mechanism of action and/or clinical efficacy but didn’t assist in RP2D decision (3), results presented and correlated with a readout of mechanism of action and/or clinical efficacy and assisted RP2D decision (4), results presented but not correlated with a readout of mechanism of action and/or clinical efficacy and didn’t assist in RP2D decision (5), results presented and not correlated with a readout of mechanism of action and/or clinical efficacy, but assisted in the RP2D decision

### Pharmacodynamic analyses reported in clinical trials

The pharmacodynamic analyses described were conducted at different levels, either at molecule, protein, transcriptome or at genome level (Table 2 and Appendix III). Molecule or protein level assessments included: (1) a change in direct readout of the mechanism of action through quantification of target protein or molecule level before and on-treatment (e.g. change in phosphorylated mesenchymal-epithelial transition factor (MET) expressions by immunohistochemical staining in tumor tissue, following MET inhibition [40]), (2) a change in indirect readout of the mechanism of action, which could be systemic (e.g., change in serum phosphate levels following Fibroblast Growth Factor Receptor (FGFR) inhibition [41, 42]) or tumor specific (e.g., Kiel antigen 67 (KI-67) immunohistochemical staining in tumor tissue at baseline versus on-treatment samples [43–45]) or (3) a change in target occupancy (e.g. cyclin-dependent kinase 7 (CDK7) protein bound versus total receptor occupancy following CDK7 inhibition [46]). The transcriptome and genome-level analyses included: (1) identification of genomic alterations at baseline, which did or did not correlate with radiological responses (e.g. response of patients with wild-type BRCA mutations at baseline treated with PARP inhibitor [47]), (2) changes in genomic profile by comparing the baseline and on-treatment profiles (e.g. DNA analysis to assess variants, mutational signatures and copy numbers pre-dose and post-dose following treatment with ATR inhibitor [48]), or (3) change in the expression of one specific gene serving as a readout of mechanism of action (e.g. change in mRNA expression of MYC following treatment with CDK9 inhibitor [49]). These analyses were performed using established molecular techniques, including Polymerase Chain Reaction (PCR), Next-Generation Sequencing (NGS) or Whole Genome Sequencing (WGS), with the choice of method guided by the characteristics of the compound and/or target. It should be noted that in this review, baseline genomic alterations were classified as pharmacodynamic biomarkers when the original trial manuscripts labeled them as such. However, genomic alterations do not represent pharmacodynamic biomarkers, as they do not reflect drug-induced biological change. Only when genomic alterations are used to track molecular change after treatment (e.g. change in genomic profile and change in ctDNA), can they be considered as pharmacodynamic [50]. All compounds and targets were sub-categorized in Table 2. Refer to Appendix III for a detailed overview of the specific compounds and targets included.

**Table 2:** Types of pharmacodynamic analyses reported in classified articles

		No PD analysis results presented	PD analysis results presented				Total
			Correlation between results and dose levels		No correlation between results and dose levels		
			Results didn’t assist RP2D	Results assisted RP2D	Results didn’t assist RP2D	Results assisted RP2D	
		#1	#2	#3	#4	#5	
*Tumor tissue*							
Change in direct readout of mechanism of action		0	5	6	0	0	11
Change in indirect readout of mechanism of action		0	1	4	0	0	5
Baseline genomic alterations*		0	2	1	2	0	5
Change in genome*		0	0	5	0	0	5
Change in transcriptome**		0	1	3	0	0	4
*Blood*							
Change in direct readout of mechanism of action		0	4	8	1	0	13
Change in indirect readout of mechanism of action		0	2	4	0	0	6
Change in percentage target occupancy		0	0	1	0	0	1
Baseline genomic alterations*		0	0	4	1	0	5
Change in genome*		0	0	1	0	0	1
Change in transcriptome**		0	1	2	0	0	3
Change in ctDNA level		0	4	6	0	0	10
Change in immune cell level		0	0	2	0	0	2
*Cerebral spinal fluid*							
Concentration drug at target site		0	1	0	0	0	1
*Skin tissue*							
Change in percentage target occupancy		0	0	1	0	0	1
*Imaging*							
Change in direct readout of mechanism of action		0	1	2	0	0	3

Study classes were as follows: (1) no results presented (2), results presented and correlated with a readout of mechanism of action and/or clinical efficacy but did not assist in RP2D decision (3), results presented and correlated with a readout of mechanism of action and/or clinical efficacy and assisted RP2D decision (4), results presented but not correlated with a readout of mechanism of action and/or clinical efficacy and did not assist in RP2D decision (5), results presented and not correlated with a readout of mechanism of action and/or clinical efficacy, but assisted in the RP2D decision. Full list of compounds per class can be found in Appendix III. PD = pharmacodynamic; RP2D = recommended phase II dose

*Genomic alterations are measured by Polymerase Chain Reaction or Next-Generation Sequencing

**Difference in gene expression by Polymerase Chain Reaction, Next-Generation Sequencing or Whole Genome Sequencing

Additional pharmacodynamic analyses included modulation of the immune (micro)environment. These were performed on a systemic level by assessing changes in circulating immune cell populations. Furthermore, changes in ctDNA were measured in correlation with radiological responses. Methods to use ctDNA, in relation to clinical responses, were: (1) a decline in one tumor specific mutation measured in ctDNA over time, (2) a decline in the total fraction of ctDNA in the cell-free DNA over time, (3) a decline in mean variant allele frequencies (VAFs) over time. It can be argued that change in ctDNA is not a pharmacodynamic endpoint, as it is often seen as an early response assessment. However, the use of ctDNA in first-in-human clinical trials is not a direct response measurement, but a molecular measurement correlated with clinical response and therefore classified as pharmacodynamic analysis in this review [51]. 

Pharmacodynamic analyses were often treated as exploratory objectives with minimal specification of methods in the clinical study protocol, while development of a fit-for-purpose robust and reproducible pharmacodynamic analysis method requires, amongst others, collaboration with multiple experts, collection of well-preserved tumor samples and adequate equipment. For example, a limited understanding of the mechanism of action of a new compound that enters a FIH clinical trial, might also result in methodological choices for pharmacodynamic analyses that ultimately limit the interpretability of the results. Therefore, pharmacodynamic analyses should not be preserved as solely exploratory objectives to optimize the utility of the analyses.

### Biological matrices reported in clinical trials

The most frequently reported biological matrix was blood. 82% of the clinical trials (*n* = 28/34) reported at least one blood-based pharmacodynamic analysis. In addition, the majority of the performed analyses were in blood (*n* = 41/76, 54%). Sixteen clinical trials (47%) included at least one pharmacodynamic analysis was tumor tissue-derived, of which fourteen trials obtained fresh tumor tissue from patients for these analyses. In thirteen trials, paired fresh tumor tissue samples were collected. The remaining three clinical trials used archival material (e.g. for analyzing baseline genomic alterations).

Change in direct readout of the mechanism of action was the most often performed type of pharmacodynamic analysis, both in tumor tissue (*n* = 11/30, 37%) and blood (*n* = 13/41, 33%), and 100% in imaging (*n* = 3/3). It is notable that three FIH trials studying an estrogen receptor (ER) degrader in breast cancer patients, used imaging as a pharmacodynamic analysis to measure changes in the direct readout of the mechanism of action. In these trials positron emission tomography (PET) scanning was used to image the change in estrogen receptors, by the radioactive tracer 18 F-fluorestradiol (FES) that binds to ER in cancer cells [44, 45, 52]. A known limitation of FES-PET is that reduction in tracer uptake may reflect drug-induced receptor occupancy rather than actual decrease in ER expression, as the tracer bind to the same ligand-binding pocket as many ER-directed agents. Therefore, FES-PET cannot differentiate between target degradation and occupancy, limiting interpretability as a pharmacodynamic marker [53, 54]. 

### Use of trial results in the RP2D decision making

All clinical trials that described a pharmacodynamic analysis in their methods section, included the results in the manuscript. Therefore, no trials were classified in the no results presented category (#1, Fig. 2).

#### Results of pharmacodynamic analyses assisted RP2D decision making

Encouragingly, half of the clinical trials that included pharmacodynamic analyses in their methods section, and therefore subjected patients to additional (invasive) procedures, did use pharmacodynamic results and reported how these results contributed to finding the RP2D and/or strengthening the chosen RP2D (*n* = 17/34, 50%, #3, Fig. 2). Note that this may be an overestimation, as the clinical trials included in this review mainly involved compounds that concluded with a RP2D. However, some clinical trials might have used the pharmacodynamic analyses in the RP2D decision making without recording as such, and therefore these results might be an underestimation.

Among the clinical trials that reported the use of pharmacodynamic results in their RP2D decision-making process, the majority performed these analyses in blood (*n* = 16/17, 94%). Tumor tissue was reported less often as biological matrix (*n* = 8/17, 45%). Moreover, of the trials that conducted blood-based pharmacodynamic analyses, 14 used these results in determining the RP2D (*n* = 14/16, 88%). If no blood-based pharmacodynamic result was assisted determining the RP2D, the determination relied on analyses performed with skin tissue and imaging [45, 46]. 

Of the clinical trials that conducted tissue-derived pharmacodynamic analyses, six used these results in the RP2D decision making (*n* = 6/8, 75%). The studies that did not report the tissue-derived pharmacodynamics, reported either blood-based [45] or imaging-based [55] pharmacodynamics in their RP2D decision. Remarkably, both these described seven pharmacodynamic analyses in their methods section. The first trial only reported results from three out of seven analyses: two blood-based and one tissue-derived. One blood-based analysis measuring a direct readout of the mechanism of action was correlated with the dose level and assisted the RP2D decision. The second clinical trial reported results from four out of seven analyses: two tissue-derived, one blood-based and one imaging-based analysis. However, the tissue-derived analyses were based on only three paired biopsies; therefore, no conclusions could be drawn. In that trial, only the imaging results assisted the RP2D decision [45]. Performing multiple analyses raises the question of how strong the readouts will correlate, and whether any form of multiple‑testing correction was applied in such situations.

Overall, the potential added value of the blood-based pharmacodynamic analyses is reflected in both quantity and quality. Blood is not only the most frequently collected biological matrix, but also the matrix most often reported to inform RP2D decision making. This likely reflects the advantages of blood-based biomarkers, as they can be sampled frequently with minimal burden. This enables more accurate assessment of changes over time and reproducibility. In addition, blood‑based biomarkers help avoid the impact of tumor heterogeneity. However, it should be taken into account that blood‑based pharmacodynamic analyses remain surrogate measures for the mechanism of action.

The types of pharmacodynamic analyses most successfully used in the RP2D decision-making was, based on the limited number of observations in this study, to assess changes in genomic and transcriptomic profile by comparing the baseline and on-treatment profiles in tissue or blood. However, the targets of these genomic/transcriptomic analyses were often not specified. Secondly, an indirect readout of the mechanism of action tested in tumor tissue also seems successful, however this cannot be determined based on this limited number of articles that used this pharmacodynamic method.

#### Results of pharmacodynamic analyses did not assist RP2D decision making

In total, 41% of the clinical trials that performed a pharmacodynamic analysis, and found a correlation of the results with the analyzed doses, did not report those results in their RP2D selection (*n* = 14/34, #2, Fig. 2).

Invasive procedures, such as tumor biopsies and cerebral spinal fluid punctures, were performed in almost half of these clinical trials (42%, *n* = 6/14), but none reported use of their correlated results in their RP2D decision-making process [40, 44, 56–59]. It is notable that the majority of the clinical trials only performed one pharmacodynamic analysis (*n* = 10/14, 71%). The advantage of using one analysis could be that it avoids the potential of ‘cherry‑picking’ the readout that most conveniently supports the intended interpretation. Most clinical trials provided explanations for not using pharmacodynamic analysis results in their RP2D decision; (1) the sample size per dose level was too small, allowing only general pharmacodynamic analyses to improve understanding of the biological activity [44, 59–61], and/or (2) the trial failed to collect an adequate number of paired blood or biopsy samples to draw definite conclusions [56, 62, 63], and/or (3) the pharmacodynamic analysis method proved to be inadequate or required further validation [44, 49, 59, 62, 64]. However, some clinical trials did not provide any explanation for not using their pharmacodynamic analysis results in their RP2D decision-making process [40, 57, 58, 65]. The determination of the RP2D was achieved in half of these clinical trials solely based on safety and/or tolerability (*n* = 7/14, 50%). Of the other seven clinical trials; four based their RP2D decision on safety, efficacy and pharmacokinetics, two trials were terminated before any conclusion could be reached and one trial included in vitro research instead of their pharmacodynamic analyses in their RP2D decision making.

Three clinical trials (9%) found no correlation with a readout of mechanism of action or efficacy in all their pharmacodynamic analyses. Unsurprisingly, none of these three studies included those results in the RP2D decision making (*n* = 3/34, #4; and *n* = 0/34, #5, Fig. 2). It should be noted that reporting pharmacodynamic results, regardless of their outcome, is important as this may prevent use of the same pharmacodynamic analysis for the same target in future research.

### Ethical perspectives

In contrast to clinical biopsies, where tissue is collected for diagnostic purposes, research biopsies are performed for scientific purposes only. Biopsies are included in clinical trial protocols because the potential scientific benefit is high. Research biopsies, however, do not directly benefit patients but are mandatory in clinical trial protocols and carry known risks [1]. Patients undergo these non-diagnostic procedures under the assumption that they will help to develop treatment options for future cancer patients and help provide insight into drug response and resistance mechanisms. The EU Clinical Trial Regulation (Article 28 CTR) [66] and FDA Informed Consent Guidance [8] regulate that a clinical trial should involve as little pain, discomfort, fear and any other foreseeable risks as possible for the patients. Furthermore, the Medical Ethics Committee evaluates the balance between optimal data collection and the potential harm to patients prospectively, under the assumption that the results of clinical trials are presented in the public domain.

Potential underreporting or inadequate use of pharmacodynamic analyses results could raise ethical concerns, as the patients are exposed to potential harm and burden (including pain, discomfort, fear and procedure related risks) of invasive non-diagnostic procedures to obtain tumor tissue without gaining the benefit of contribution to public knowledge. For example, in this study, three clinical trials were excluded as they did not report any pharmacodynamic analyses in their methods and results section, but they did describe biopsy sampling in their clinical trial protocol (*n* = 3/51 6%) [30, 33, 38]. In addition, multiple clinical trials did perform biopsies to collect tumor tissue and found a correlation of their results to the dose level, but did not describe whether or how they used their data in their RP2D decision-making process [40, 44, 45, 55–57, 59]. In these clinical trials it may be questioned whether the risk and burden of these biopsies were sufficiently in balance with their merits for the trial. Also, subjecting patients to invasive procedures while a definite conclusion cannot be drawn, due to a (planned) limited sample size or lack of validated pharmacodynamic analysis method, raises ethical concerns; particularly since these limitations may be identifiable before initiating the trial.

This review should be interpreted in light of some limitations. The small sample size and selection of only published clinical trials may introduce potential selection bias. However, this review evaluated to what extent pharmacodynamic analyses are used in the determination of the RP2D in recent FIH trials of small-molecule targeted agents. In addition, the inclusion of pharmacodynamic analyses relied on the original author’s labeling. Therefore, baseline genomic alterations were considered as pharmacodynamic analyses in this review, although these biomarkers represent prediction instead of pharmacodynamics. This may have led to an overestimation of the extent to which pharmacodynamic data were available in the investigated trials.

## Conclusion

As targeted agents remain the dominant class in drug development, the use of pharmacodynamic analyses in RP2D decisions is increasing [21]. Encouragingly, half of the clinical trials in this study incorporated and reported their pharmacodynamic analysis results into their RP2D determination. Nevertheless, underreporting and inadequate use of pharmacodynamic analysis results persists, with a potential inadequate balance between the risk and benefit for patients on which we rely for advancing the field. Minimally invasive pharmacodynamic analyses, such as blood-based analyses, should be considered more to replace invasive procedures such as research biopsies, as these pharmacodynamic results are more often used to assist the RP2D decision-making process. Finally, it is crucial to carefully prepare and choose a fit-for-purpose pharmacodynamic analysis method before study initiation, select an appropriate sample size and validate pharmacodynamic assays to avoid subjecting patients to invasive procedures without obtaining meaningful results.

## Supplementary Information

Below is the link to the electronic supplementary material.

Supplementary Material 1

## Data Availability

All data analyzed in this study were obtained from public sources.
