# Supplementary material for: The use of pharmacodynamic results for recommended phase II decision making in oncology clinical trials
Source: Cancer Chemother Pharmacol. 2026 Jul 6;96(1):70. doi: 10.1007/s00280-026-04924-7 (PMC13337676; doi:10.1007/s00280-026-04924-7)
Supplement: Supplementary file 1 — Supplementary Material 1 [file 280_2026_4924_MOESM1_ESM.pdf]

# The use of pharmacodynamic results for recommended phase II decision making in oncology clinical trials

A.C. Kanhailal[1] *, M.J.J. Lucassen[1] *, T. Schutte[1], W. Zwart[2,3,4], A.D.R. Huitema,[5,6,7], N. Steeghs[1,8]

***Both authors contributed equally**

**Affiliations**

1. Department of Medical Oncology, The Netherlands Cancer Institute, Amsterdam, The Netherlands.
2. Oncode Institute, Utrecht, the Netherlands
3. Division of Oncogenomics, The Netherlands Cancer Institute, Amsterdam, The Netherlands
4. Department of Biomedical Engineering, Eindhoven University of Technology, Eindhoven, The Netherlands
5. Department of Pharmacy & Pharmacology, The Netherlands Cancer Institute, Amsterdam, The Netherlands.
6. Princess Máxima Center for Pediatric Oncology, Utrecht, The Netherlands.
7. Department of Clinical Pharmacy, University Medical Center Utrecht, Utrecht University, Utrecht, The Netherlands.
8. Department of Medical Oncology, University Medical Center Utrecht, Utrecht University, Utrecht, The Netherlands.

# Appendix I: Search and data analysis

## Research question

How do pharmacodynamic analyses assist in the determination of the recommended phase II dose of single agent small molecules in First-in-Human oncology clinical trials?

## Search strategy

The full search term was defined as:

(((((neoplasms[mesh terms] OR neoplasm*[tiab] OR tumor*[tiab] OR tumour*[tiab] OR cancer*[tiab] OR malign*[tiab] OR carcino*[tiab] OR metasta*[tiab] OR sarco*[tiab] OR melano*[tiab] OR oncolog*[tiab]) AND ("phase 1"[tiab] OR "phase one"[tiab] OR "phase I"[tiab] OR "phase1"[tiab] OR "phaseI"[tiab] OR "phase 0"[tiab] OR "Clinical Trials, Phase I as Topic"[MeSH Terms] OR "dose escalation"[tiab] OR "first in human"[tiab] OR "first in man"[tiab] OR "First in patient"[tiab])) AND (pharmacodynamics[mesh terms] OR pharmacodynamic*[tiab] OR biopsies[mesh terms] OR biops*[tiab] OR immunohistochemistry[mesh terms] OR immunohistochemi*[tiab] OR "target engagement"[tiab] OR biomarker*[tiab] OR biomarkers[mesh terms] OR "response marker*"[tiab] OR "resistance marker*"[tiab] OR pharmacogenetics[mesh terms] OR pharmacogenetic*[tiab] OR "translational science, biomedical"[mesh terms] OR pharmacogenomics[mesh terms] OR pharmacogenomic*[tiab] OR transcriptomic*[tiab] OR proteomics[mesh terms] OR proteomic*[tiab] OR "receptors, drug"[mesh terms] OR "Molecular Diagnostic Techniques"[mesh terms] OR "Molecular Diagnostic Technique*"[tiab] OR "circulating tumor DNA"[mesh terms] OR "circulating tumor DNA"[tiab] OR ctDNA[tiab] OR "Enzyme-Linked Immunosorbent Assay"[mesh terms] OR "Enzyme-Linked Immunosorbent Assay"[tiab] OR ELISA[tiab] OR immunoassay*[tiab] OR immunocytochemi*[tiab] OR immunofluorescen*[tiab] OR fluorescen*[tiab] OR "cell-free nucleic acids"[Mesh terms] OR "cell-free DNA" [tiab] OR cfDNA [tiab] OR "Transcriptome Regulation"[MeSH terms] OR "Transcriptome"[tiab] OR "Neoplasms/metabolism"[MeSH] OR "Neoplasms/pathology"[MeSH])) AND ("Small molecule inhibitor*"[Title/Abstract] OR "Targeted Therap*"[Title/Abstract] OR "Protein Kinase Inhibitors"[MeSH Terms] OR "Tyrosine Kinase Inhibitor*"[Title/Abstract] OR "Tyrosine protein kinase*"[Title/Abstract] OR "EGFR inhibitor*"[Title/Abstract] OR "epidermal growth factor receptor inhibitor*"[Title/Abstract] OR "ALK inhibitor*"[Title/Abstract] OR "anaplastic lymphoma kinase inhibitor*"[Title/Abstract] OR "ROS1 inhibitor*"[Title/Abstract] OR "BCR-ABL inhibitor*"[Title/Abstract] OR "VEGFR inhibitor*"[Title/Abstract] OR "vascular endothelial growth factor receptor inhibitor*"[Title/Abstract] OR "RET inhibitor*"[Title/Abstract] OR "TRK inhibitor*"[Title/Abstract] OR "NTRK inhibitor*"[Title/Abstract] OR "tropomyosin receptor kinase inhibitor*"[Title/Abstract] OR "HER2 inhibitor*"[Title/Abstract] OR "HER3 inhibitor*"[Title/Abstract] OR "human epidermal growth factor receptor inhibitor*"[Title/Abstract] OR "Serine/Threonine kinase inhibitor*"[Title/Abstract] OR "BRAF inhibitor*"[Title/Abstract] OR "B-RAF inhibitor*"[Title/Abstract] OR "RAF inhibitor*"[Title/Abstract] OR "MEK inhibitor*"[Title/Abstract] OR "mitogen-activated protein kinase inhibitor*"[Title/Abstract] OR "MTOR inhibitor*"[Title/Abstract] OR "mammalian target of rapamycin inhibitor*"[Title/Abstract] OR "ERK inhibitor*"[Title/Abstract] OR "extracellular signal regulated kinase inhibitor*"[Title/Abstract] OR "CDK4/6 inhibitor*"[Title/Abstract] OR "cyclin dependent kinase 4/6 inhibitor*"[Title/Abstract] OR "proteasome inhibitors"[MeSH Terms] OR "proteasome inhibitor*"[Title/Abstract] OR "Poly(ADP-ribose) Polymerase Inhibitors"[MeSH Terms] OR "Poly(ADP-ribose) Polymerase Inhibitor*"[Title/Abstract] OR "PARP inhibitor*"[Title/Abstract] OR "Hedgehog pathway inhibitor*"[Title/Abstract] OR "IDH inhibitor*"[Title/Abstract] OR "Isocitrate dehydrogenase 1 inhibitor*"[Title/Abstract] OR "Janus Kinase Inhibitors"[MeSH Terms] OR "JAK inhibitor*"[Title/Abstract] OR "Janus Kinase Inhibitor*"[Title/Abstract] OR "BTK inhibitor*"[Title/Abstract] OR "Bruton's tyrosine kinase inhibitor*"[Title/Abstract] OR "Protein kinase inhibitor*"[Title/Abstract] OR "PDGFR inhibitor*"[Title/Abstract] OR "platelet derived growth factor receptor inhibitor*"[Title/Abstract] OR "KIT inhibitor*"[Title/Abstract] OR "FLT3 inhibitor*"[Title/Abstract] OR "fms-like tyrosine kinase 3 inhibitor*"[Title/Abstract] OR "Histone Deacetylase Inhibitors"[MeSH Terms] OR "HDAC inhibitor*"[Title/Abstract] OR "histone deacetylase inhibitor*"[Title/Abstract] OR "FGFR

inhibitor*"[Title/Abstract] OR "fibroblast growth factor inhibitor*"[Title/Abstract] OR "MET inhibitor*"[Title/Abstract] OR "mesenchymal-epithelial transition factor inhibitor*"[Title/Abstract] OR "SMO inhibitor*"[Title/Abstract] OR "smoothened inhibitor*"[Title/Abstract] OR "Tie2 inhibitor*"[Title/Abstract] OR "Pi3k inhibitor*"[Title/Abstract] OR "phosphatidylinositol 3-kinases inhibitor*"[Title/Abstract] OR "AKT inhibitor*"[Title/Abstract] OR "BCL2 inhibitor*"[Title/Abstract] OR "ezh2 inhibitor*"[Title/Abstract] OR "enhancer of zeste homolog 2 inhibitor*"[Title/Abstract] OR "DNMT inhibitor*"[Title/Abstract] OR "CXCR4 inhibitor*"[Title/Abstract] OR "CSF1R inhibitor*"[Title/Abstract] OR "colony stimulating factor 1 receptor inhibitor*"[Title/Abstract] OR "XPO1 inhibitor*"[Title/Abstract] OR "exportin 1 inhibitor*"[Title/Abstract] OR "androgen receptor inhibitor*"[Title/Abstract] OR "AR inhibitor*"[Title/Abstract] OR "CYP17A1 inhibitor*"[Title/Abstract] OR "GnRH receptor antagonist*"[Title/Abstract] OR "gonadotropin-releasing hormone antagonist*"[Title/Abstract] OR "aromatase inhibitor*"[Title/Abstract] OR "aromatase inhibitors"[MeSH Terms] OR "ER inhibitor*"[Title/Abstract] OR "estrogen receptor inhibitor*"[Title/Abstract] OR "estrogen antagonists"[MeSH Terms] OR "methyltransferase inhibitor*"[Title/Abstract] OR "multi-kinase inhibitor*"[Title/Abstract] OR "KRAS inhibitor*"[Title/Abstract] OR "RAS inhibitor*"[Title/Abstract] OR "RXR agonist*"[Title/Abstract] OR "RXR activator*"[Title/Abstract] OR "retinoid X receptor agonist*"[Title/Abstract] OR "somatostatin analog*"[Title/Abstract] OR "HIF-2A inhibitor*"[Title/Abstract] OR "hypoxia-inducible factor 2 alpha inhibitor*"[Title/Abstract] OR "CRBN modulator*"[Title/Abstract] OR "cereblon modulator*"[Title/Abstract])) NOT ("combination"[Title] OR "combined"[Title])) AND ((2022/01/01:2025/07/01[pdat]) AND (dutch[Filter] OR english[Filter]

## Analysis description

The extracted data was semantically analyzed using an inductive approach. This meant that an explicit description was used to categorize an pharmacodynamic analysis, e.g. only if the author of a manuscript described ‘pharmacodynamic analyses’ it was used as such. An inductive approach meant that categories were formed, such as ‘change in direct readout of mechanism of action’, based on the specific analyses that were found. Using the semantic analysis with an inductive approach, the explicit data determined the categories that were formed.

Appendix II: Characteristics of included clinical trials

| **First author** | **Year of publication** | **Compound discontinued after Phase I** | **Indicated tumor type(s) tested in clinical trial** | **Drug class** | **Number of pharmacodynamic analyses** | **Type of pharmacodynamic analyses** | *Ref* |
|---|---|---|---|---|---|---|---|
| Bauer | 2022 | No | Solid tumors | TAM inhibitor | 1 | Change in direct readout of mechanism of action | *[1]* |
| Bardia | 2022 | No | Breast cancer | ER degrader | 1 | Change in direct readout of mechanism of action | *[2]* |
| Bardia | 2023 | No | Breast cancer | ER degrader | 7 | Change in direct readout of mechanism of action [a]<br>Change in indirect readout of mechanism of action [b]<br>Change in genome<br>Change in transcriptome<br>Baseline genomic alterations | *[3]* |
| Chandarlapaty | 2023 | No | Breast cancer | ER degrader | 4 | Change in direct readout of mechanism of action [a]<br>Change in indirect readout of mechanism of action<br>Change in ctDNA level | *[4]* |
| Coombes | 2023 | No | Breast cancer | CDK-7 inhibitor | 3 | Change in direct biomarker [a]<br>Change in ctDNA level | *[5]* |

| First author | Year of publication | Compound discontinued after Phase I | Indicated tumor type(s) tested in clinical trial | Drug class | Number of pharmacodynamic analyses | Type of pharmacodynamic analyses | *Ref* |
|---|---|---|---|---|---|---|---|
| Diamond | 2022 | No | Solid tumors or Non Hodgkin lymphoma | CDK9 inhibitor | 1 | Change in transcriptome | *[6]* |
| Doi | 2024 | No | Solid tumors | AKT inhibitor | 7 | Change in direct readout of mechanism of action [a,c]<br>Baseline genomic alterations [b]<br>Change in genome [b] | *[7]* |
| Garmezy | 2024 | Yes | Solid tumors | FGFR inhibitor | 4 | Change indirect biomarker expression [a]<br>Change in direct biomarker expression<br>Change in ctDNA level | *[8]* |
| Garralda | 2024 | Yes | Solid tumors | CDK7 inhibitor | 2 | Change in percentage target occupancy [a] | *[9]* |
| Gelderblom | 2024 | No | Solid tumors or Tenosynovial Giant Cell Tumor | CSF1R inhibitor | 2 | Change in immune cell level<br>Change in direct readout of mechanism of action | *[10]* |
| Guo | 2024 | No | Solid tumors | TGFβ inhibitor | 1 | Change in direct readout of mechanism of action | *[11]* |
| Hamilton | 2023 | No | Solid tumors | BET inhibitor | 4 | Change in genome [a]<br>Change in transcriptome [a] | *[12]* |

| First author | Year of publication | Compound discontinued after Phase I | Indicated tumor type(s) tested in clinical trial | Drug class | Number of pharmacodynamic analyses | Type of pharmacodynamic analyses | *Ref* |
|---|---|---|---|---|---|---|---|
| Hamilton | 2025 | No | Breast cancer | CERAN/SERD | 2 | Change in ctDNA level<br>Change in indirect readout of mechanism of action | *[13]* |
| Harding | 2023 | Yes | Solid tumors | FGFR inhibitor | 1 | Change in direct readout of mechanism of action | *[14]* |
| Hilton | 2022 | No | Lymphoma and Solid tumors | BRD4 inhibitor | 2 | Change in transcriptome<br>Change in indirect readout of mechanism of action | *[15]* |
| Janku | 2024 | No | Solid tumors | Pan-RAF inhibitor | 1 | Change in direct readout of mechanism of action | *[16]* |
| Kang | 2023 | No | Solid tumors | VEGFR/CSF1R inhibitor | 1 | Change in direct readout of mechanism of action | *[17]* |
| Kristeleit | 2023 | Yes | Solid tumors | CHK1 inhibitor | 2 | Baseline genomic alterations [a] | *[18]* |
| Kim | 2025 | No | Solid tumors | PARP inhibitor | 4 | Change in direct readout of mechanism of action [a]<br>Baseline genomic alterations [a] | *[19]* |
| Li | 2024 | No | Solid tumors | RET inhibitor | 1 | Change in ctDNA level | *[20]* |
| Ma | 2022 | No | NSCLC | ALK inhibitor | 1 | Baseline genomic alterations | *[21]* |

| First author | Year of publication | Compound discontinued after Phase I | Indicated tumor type(s) tested in clinical trial | Drug class | Number of pharmacodynamic analyses | Type of pharmacodynamic analyses | *Ref* |
|---|---|---|---|---|---|---|---|
| Pipero-Neumann | 2023 | No | Uveal melanoma | PKC inhibitor | 3 | Change in direct biomarker expression [a]<br>Change in transcriptome | *[22]* |
| Pruis | 2023 | Yes | NSCLC | MET/OCT-2 inhibitor | 3 | Change in direct readout of mechanism of action<br>Change in indirect readout of mechanism of action [b] | *[23]* |
| Ruan | 2025 | No | NSCLC | RET/VEGFR-2/EGFR/FGFR1-3 inhibitor | 1 | Baseline genomic alterations | *[24]* |
| Schreiber | 2023 | No | Solid tumors | HDAC inhibitor | 1 | Change in direct readout of mechanism of action | *[25]* |
| Shi | 2024 | No | NSCLC | ALK inhibitor | 1 | Change in ctDNA level | *[26]* |
| Subbiah | 2022 | No | Solid tumors | FGFR1-3 inhibitor | 3 | Change in direct readout of mechanism of action [b]<br>Change in indirect readout of mechanism of action | *[27]* |
| Tsimberidou | 2025 | No | Solid tumors | STAT3 inhibitor | 2 | Change in direct readout of mechanism of action<br>Baseline genomic alterations | *[28]* |

| First author | Year of publication | Compound discontinued after Phase I | Indicated tumor type(s) tested in clinical trial | Drug class | Number of pharmacodynamic analyses | Type of pharmacodynamic analyses | *Ref* |
|---|---|---|---|---|---|---|---|
| Wang | 2023 | No | Melanoma | MEK inhibitor | 1 | Change in ctDNA level | *[29]* |
| Yap | 2023 | No | Solid tumors | ATR inhibitor | 3 | Change in ctDNA level<br>Change in indirect readout of mechanism of action<br>Change in genome | *[30]* |
| Yap | 2024 | Yes | Solid tumors | ATR inhibitor | 3 | Change in indirect readout of mechanism of action<br>Change in immune cell level<br>Change in ctDNA level | *[31]* |
| Yap | 2024 | No | Solid tumors | TGFβ inhibitor | 1 | Change in direct readout of mechanism of action | *[32]* |
| Zhang | 2023 | No | Breast cancer | HER2 inhibitor | 1 | Concentration drug at target site | *[33]* |
| Zhao | 2025 | No | NSCLC | ALK/ROS1 inhibitor | 1 | Change in ctDNA level | *[34]* |

Table 1. Characteristics of included clinical trials.

AKT = protein kinase B; ALK = anaplastic lymphoma kinase; ATM = ataxia-telangiectasia mutated; ATR = ataxia telangiectasia and Rad3-related protein; BET = bromodomain and extra-terminal; BRD = bromodomain protein; CDK = cyclin-dependent kinase; CERAN = complete estrogen receptor antagonist; CHK = checkpoint kinase; ctDNA = circulating tumor DNA; CSF1R = colony stimulating factor 1 receptor; EGFR = epidermal growth factor receptor; ER = estrogen receptor; FGFR = fibroblast growth factor receptor; HDAC = histone-deacetylase; HER = human epidermal growth factor receptor; MEK = mitogen-activated protein kinase; MET = mesenchymal epithelial transition receptor; NSCLC; non-small cell lung cancer OCT = organic cation transporter; PARP = poly (ADP-ribose) polymerase; PKC = protein kinase C; RAF = rapidly accelerated fibrosarcoma; RET = rearranged during transfection; ROS = ROS proto-oncogene ; SERD = selective estrogen receptor degrader; STAT = signal transducer and activator of transcription; TAM = Tyro3, Axl and Mer; TGFβ =

transforming growth factor-beta; VEGFR = vascular endothelial growth factor receptor.

a Two biomatrices

b Two analyses

c Three analyses

## Appendix III: Types of pharmacodynamic analyses reported in classified articles

| | | No PD analysis results presented | PD analysis results presented | | | | |
|---|---|---|---|---|---|---|---|
| | | | Correlation between results and dose levels | | No correlation between results and dose levels | | |
| | | | Results didn't assist RP2D | Results assisted RP2D | Results didn't assist RP2D | Results assisted RP2D | |
| | | #1 | #2 | #3 | #4 | #5 | Total |
| *Tumor tissue* | | | | | | | |
| Change in direct readout of mechanism of action | | 0 | 5 | 6 | 0 | 0 | 11 |
| *Compound* | *Target* | | | | | | |
| MET/OCT-2 inhibitor | Total MET, p-MET | | X | | | | |
| PKC inhibitor | p-PKCδ, p-MARCKS | | X | | | | |
| STAT3 inhibitor | Anti-p-tyrosine STAT3 | | X | | | | |
| pan-RAF kinase inhibitor | DUSP6 | | X | | | | |
| ER degrader | ER and PR | | X | X | | | |
| PARP inhibitor | PARP | | | X | | | |
| CDK 7 inhibitor | p-Poll, p-CDK1/2/3 | | | X | | | |
| FGFR inhibitor | p-ERK, total ERK | | | X | | | |
| | DUSP6 | | | X | | | |
| AKT inhibitor | AKT, pan-AKT | | | X | | | |
| Change in indirect readout of mechanism of action | | 0 | 1 | 4 | 0 | 0 | 5 |
| *Compound* | *Target* | | | | | | |
| ER degrader | Ki67 | | X | X | | | |
| | Optional IHC without target specified | | | X | | | |
| CERAN /SERD | Ki67 | | | X | | | |
| ATR inhibitor | p-KAP1, ɣH2AX | | | X | | | |
| Baseline genomic alterations* | | 0 | 2 | 1 | 2 | 0 | 5 |
| *Compound* | *Target* | | | | | | |
| multi-kinase RET/VEGFR/EGFR/ FGFR1-3 inhibitor | TP53, SMARCA4, SMARCB1, EZH2 | | X | | | | |
| STAT3 inhibitor | Exploratory analysis without target specified, p53 | | X | | | | |
| PARP inhibitor | BRCA, HRR | | | X | | | |
| CHK1 inhibitor | MSI, TMB | | | | X | | |
| ALK inhibitor | ALK | | | | X | | |
| Change in genome* | | 0 | 0 | 5 | 0 | 0 | 5 |
| *Compound* | *Target* | | | | | | |
| AKT inhibitor | PIK3CA, AKT1 | | | X | | | |
| | Genomic abnormalities | | | X | | | |
| ATR inhibitor | Genomic abnormalities | | | X | | | |
| ER degrader | No target specified | | | X | | | |

| | | | No PD analysis results presented | PD analysis results presented | | | | |
|---|---|---|---|---|---|---|---|---|
| | | | | Correlation between results and dose levels | | No correlation between results and dose levels | | |
| | | | | Results didn't assist RP2D | Results assisted RP2D | Results didn't assist RP2D | Results assisted RP2D | |
| | | | #1 | #2 | #3 | #4 | #5 | Total |
| | BET inhibitor | No target specified | | | X | | | |
| Change in transcriptome** | | | 0 | 1 | 3 | 0 | 0 | 4 |
| | *Compound* | *Target* | | | | | | |
| | PKC inhibitor | RasGRP3 | | X | | | | |
| | ER degrader | No target specified | | | X | | | |
| | BET inhibitor | CCR2, HEXIM1 | | | X | | | |
| | AKT inhibitor | AKT1, AKT2, AKT3, PIK3C2B, PHLDA1, ACTB | | | X | | | |
| *Blood* | | | | | | | | |
| Change in direct readout of mechanism of action | | | 0 | 4 | 8 | 1 | 0 | 13 |
| | *Compound* | *Target* | | | | | | |
| | VEGFR and CSF1R inhibitor | VEGFR2, VEGF and CSF1 | | X | | | | |
| | PKC inhibitor | PKC substrate protein | | X | | | | |
| | TAM inhibitor | VEGF-A and VEGF-R2 | | X | | | | |
| | FGFR inhibitor | FGF19 | | X | | | | |
| | FGFR1-3 inhibitor | p-FGFR2 alfa | | | X | | | |
| | | FGF23 | | | X | | | |
| | PARP inhibitor | PAR | | | X | | | |
| | AKT inhibitor | p-PRAS40 | | | X | | | |
| | HDAC inhibitor | Ac-H2K27, Ac-H3K9 and Ac-lysine | | | X | | | |
| | CDK7 inhibitor | p-PoLII | | | X | | | |
| | CSF1R inhibitor | CSF1 | | | X | | | |
| | TGFβ inhibitor | pSMAD2/3 | | | X | | | |
| | | Serum TGFβ-1 | | | | X | | |
| Change in indirect readout of mechanism of action | | | 0 | 2 | 4 | 0 | 0 | 6 |
| | *Compound* | *Target* | | | | | | |
| | MET/OCT-2 inhibitor | Creatinine | | X | | | | |
| | | Tryptophan, kynurenine | | X | | | | |
| | ATR inhibitor | p-H2AX, γH2AX | | | X | | | |
| | FGFR1 inhibitor | Phosphate | | | X | | | |
| | FGFR1-3 inhibitor | Phosphate | | | X | | | |
| | Bromodomain protein 4 inhibitor | MCP-1, MMP-9 | | | X | | | |
| Change in percentage target occupancy | | | 0 | 0 | 1 | 0 | 0 | 1 |
| | *Compound* | *Target* | | | | | | |
| | CDK7 inhibitor | CDK7 protein bound by study drug | | | X | | | |
| Baseline genomic alterations* | | | 0 | 0 | 4 | 1 | 0 | 5 |
| | *Compound* | *Target* | | | | | | |
| | PARP inhibitor | BRCA, HHR | | | X | | | |

| | | | No PD analysis results presented | PD analysis results presented | | | | |
|---|---|---|---|---|---|---|---|---|
| | | | | Correlation between results and dose levels | | No correlation between results and dose levels | | |
| | | | | Results didn't assist RP2D | Results assisted RP2D | Results didn't assist RP2D | Results assisted RP2D | |
| | | | #1 | #2 | #3 | #4 | #5 | Total |
| | AKT inhibitor | PIK3CA, AKT | | | X | | | |
| | | AKT1, AKT2 | | | X | | | |
| | ER degrader | ESR1 | | | X | | | |
| | CHK1 inhibitor | MSI, TMB | | | | X | | |
| Change in genome* | | | 0 | 0 | 1 | 0 | 0 | 1 |
| | *Compound* | *Target* | | | | | | |
| | BET inhibitor | No target specified | | | X | | | |
| Change in transcriptome** | | | 0 | 1 | 2 | 0 | 0 | 3 |
| | *Compound* | *Target* | | | | | | |
| | CDK9 inhibitor | MYC, MCL1, PCNA | | X | | | | |
| | BET inhibitor | No target specified | | | X | | | |
| | Bromodomain protein 4 inhibitor | BRD4 target gene | | | X | | | |
| Change in ctDNA level | | | 0 | 4 | 6 | 0 | 0 | 10 |
| | *Compound* | *Target* | | | | | | |
| | ALK inhibitor | Amount of ctDNA | | X | | | | |
| | ALK/ROS1 inhibitor | Amount of ctDNA | | X | | | | |
| | ER degrader | Amount of ctDNA | | X | | | | |
| | RET inhibitor | Amount of ctDNA | | X | | | | |
| | ATR inhibitor | Amount of ctDNA | | | X | | | |
| | CDK 7 inhibitor | Amount of ctDNA | | | X | | | |
| | MEK inhibitor | Amount of ctDNA | | | X | | | |
| | FGFR inhibitor | Amount of ctDNA | | | X | | | |
| | FGFR inhibitor | Amount of ctDNA | | | X | | | |
| | CERAN and SERD | Amount of ctDNA | | | X | | | |
| | CERAN and SERD | Number of ESR1 variant mutations | | | X | | | |
| Change in immune cell level | | | 0 | 0 | 2 | 0 | 0 | 2 |
| | *Compound* | *Target* | | | | | | |
| | ATR inhibitor | 43 immune cell subsets | | | X | | | |
| | CSF1R inhibitor | Number of monocytes | | | X | | | |
| *Cerebral spinal fluid* | | | | | | | | |
| Concentration drug at target site | | | 0 | 1 | 0 | 0 | 0 | 1 |
| | *Compound* | *Target* | | | | | | |
| | HER2 inhibitor | Drug and active metabolite | | X | | | | |
| *Skin tissue* | | | | | | | | |
| Change in percentage target occupancy | | | 0 | 0 | 1 | 0 | 0 | 1 |
| | *Compound* | *Target* | | | | | | |
| | CDK 7 inhibitor | CDK7 protein bound by study-drug | | | X | | | |
| *Imaging* | | | | | | | | |
| Change in direct readout of mechanism of action | | | 0 | 1 | 2 | 0 | 0 | 3 |

| | | No PD analysis results presented | PD analysis results presented | | | | |
|---|---|---|---|---|---|---|---|
| | | | Correlation between results and dose levels | | No correlation between results and dose levels | | |
| | | | Results didn’t assist RP2D | Results assisted RP2D | Results didn’t assist RP2D | Results assisted RP2D | |
| | | #1 | #2 | #3 | #4 | #5 | Total |
| *Compound* | *Target* | | | | | | |
| ER degrader | FES-PET | | X | X | | | |

Table 1. Types of pharmacodynamic analyses used in classified articles. (1) no results presented, (2) results presented and correlated with a readout of mechanism of action and/or clinical efficacy but did not assist in RP2D decision, (3) results presented and correlated with a readout of mechanism of action and/or clinical efficacy and assisted RP2D decision, (4) results presented but not correlated with a readout of mechanism of action and/or clinical efficacy and did not assist in RP2D decision, (5) results presented and not correlated with a readout of mechanism of action and/or clinical efficacy, but assisted in the RP2D decision. Categories are sub classified per compound and target that was used in the specified pharmacodynamic analysis.

* Genomic alterations are measured by Polymerase Chain Reaction or Next-Generation Sequencing.

**Difference in gene expression by Polymerase Chain Reaction, Next-Generation Sequencing or Whole Genome Sequencing.

Ac- = acetylated; ACTB = actin beta; AKT = protein kinase B; ALK = anaplastic lymphoma kinase; ATM = ataxia-telangiectasia mutated; ATR = ataxia telangiectasia and Rad3-related protein; BET = bromodomain and extra-terminal; BRCA = breast cancer gene; BRD = bromodomain protein; CCR = C-C motif chemokine receptor; CDK = cyclin-dependent kinase; CERAN = complete estrogen receptor antagonist; CHK = checkpoint kinase; ctDNA = circulating tumor DNA; CSF = colony stimulating factor; CSF1R = colony stimulating factor 1 receptor; DUSP = dual specificity phosphatase; EGFR = epidermal growth factor receptor; ER = estrogen receptor; ESR = estrogen receptor gene; EZH = enhancer of zeste homolog; FES-PET = fluorine 18 labelled fluoroestradiol positron emission tomography; FGF = fibroblast growth factor; FGFR = fibroblast growth factor receptor; H2AX= H2A histone family member X; H2K; histone H2A lysine; HDAC = histone-deacetylase; HER = human epidermal growth factor receptor; HEXIM = hexamethylene bis-acetamide inducible protein; HRR = homologous recombination repair; IHC = immunohistochemistry; KAP = KRAB-associated protein; Ki67 = antigen kiel 67; MARCKS = myristoylated alanine-rich C-kinase substrate; MCL = myeloid cell leukemia; MCP = monocyte chemoattractant protein; MEK = mitogen-activated protein kinase; MET = mesenchymal epithelial transition receptor; MMP = matrix metalloproteinase; MSI = microsatellite instability; MYC = myelocytomatosis viral oncogene; NSCLC; non-small cell lung cancer OCT = organic cation transporter; p- = phosphorylated; p53 = protein 53; PAR = poly ADP ribose; PARP = poly (ADP-ribose) polymerase; PCNA = proliferation cell nuclear antigen; PD = pharmacodynamic; PHLDA1 = pleckstrin homology-like domain family A member 1; PIK3CA/PIK3CB = phosphoinositide 3-kinase catalytic subunit alpha/beta; PKC = protein kinase C; PoL II = RNA polymerase II; PR = progesterone receptor; PRAS = proline-rich Akt substrate; RAF = rapidly accelerated fibrosarcoma; RasGRP = Ras guanyl-releasing protein; RET = rearranged during transfection; ROS = ROS proto-oncogene; RP2D = recommended phase II dose; SERD = selective estrogen receptor degrader; SMARCA4/SMARCB1 = SWI/SNF-related, matrix associated, actin dependent regulator of chromatin, subfamily A/B, member 4/1; STAT = signal transducer and activator of transcription; TAM = Tyro3, Axl and Mer; TGFβ = transforming growth factor-beta; TMB = tumor mutational burden; TP = tumor protein; VEGFR = vascular endothelial growth factor receptor.

References

1. Bauer T, Cho BC, Heist R, Bazhenova L, Werner T, Goel S, et al. First-in-human phase 1/1b study to evaluate sitravatinib in patients with advanced solid tumors. Invest New Drugs. 2022;40(5):990-1000.
2. Bardia A, Chandarlapaty S, Linden HM, Ulaner GA, Gosselin A, Cartot-Cotton S, et al. AMEERA-1 phase 1/2 study of amcenestrant, SAR439859, in postmenopausal women with ER-positive/HER2-negative advanced breast cancer. Nature Communications. 2022;13(1):4116.
3. Bardia A, Mayer I, Winer E, Linden HM, Ma CX, Parker BA, et al. The oral selective estrogen receptor degrader GDC-0810 (ARN-810) in postmenopausal women with hormone receptor-positive HER2-negative (HR + /HER2 -) advanced/metastatic breast cancer. Breast Cancer Res Treat. 2023;197(2):319-31.
4. Chandarlapaty S, Dickler MN, Perez Fidalgo JA, Villanueva-Vázquez R, Giltnane J, Gates M, et al. An Open-label Phase I Study of GDC-0927 in Postmenopausal Women with Locally Advanced or Metastatic Estrogen Receptor-Positive Breast Cancer. Clin Cancer Res. 2023;29(15):2781-90.
5. Coombes RC, Howell S, Lord SR, Kenny L, Mansi J, Mitri Z, et al. Dose escalation and expansion cohorts in patients with advanced breast cancer in a Phase I study of the CDK7-inhibitor samuraciclib. Nat Commun. 2023;14(1):4444.
6. Diamond JR, Boni V, Lim E, Nowakowski G, Cordoba R, Morillo D, et al. First-in-Human Dose-Escalation Study of Cyclin-Dependent Kinase 9 Inhibitor VIP152 in Patients with Advanced Malignancies Shows Early Signs of Clinical Efficacy. Clin Cancer Res. 2022;28(7):1285-93.
7. Doi T, Takahashi S, Aoki D, Yonemori K, Hara H, Hasegawa K, et al. A first-in-human phase I study of TAS-117, an allosteric AKT inhibitor, in patients with advanced solid tumors. Cancer Chemother Pharmacol. 2024;93(6):605-16.
8. Garmezy B, Borad MJ, Bahleda R, Perez CA, Chen LT, Kato S, et al. A Phase I Study of KIN-3248, an Irreversible Small-molecule Pan-FGFR Inhibitor, in Patients with Advanced FGFR2/3-driven Solid Tumors. Cancer Res Commun. 2024;4(4):1165-73.
9. Garralda E, Schram AM, Bedard PL, Schwartz GK, Yuen E, McNeely SC, et al. A Phase I Dose-Escalation Study of LY3405105, a Covalent Inhibitor of Cyclin-Dependent Kinase 7, Administered to Patients With Advanced Solid Tumors. Oncologist. 2024;29(1):e131-e40.
10. Gelderblom H, Razak AA, Taylor MH, Bauer TM, Wilky B, Martin-Broto J, et al. CSF1R Inhibition in Patients with Advanced Solid Tumors or Tenosynovial Giant Cell Tumor: A Phase I Study of Vimseltinib. Clin Cancer Res. 2024;30(18):3996-4004.
11. Guo Y, Wang Z, Zhou H, Pan H, Han W, Deng Y, et al. First-in-human study of GFH018, a small molecule inhibitor of transforming growth factor-β receptor I inhibitor, in patients with advanced solid tumors. BMC Cancer. 2024;24(1):444.
12. Hamilton EP, Wang JS, Oza AM, Patel MR, Ulahannan SV, Bauer T, et al. First-in-human Study of AZD5153, A Small-molecule Inhibitor of Bromodomain Protein 4, in Patients with Relapsed/Refractory Malignant Solid Tumors and Lymphoma. Mol Cancer Ther. 2023;22(10):1154-65.
13. Hamilton EP, Patel MR, Borges VF, Meisel JL, Okera M, Alemany CA, et al. Palazestrant, a novel oral Complete Estrogen Receptor Antagonist (CERAN) and Selective Estrogen Receptor Degrader (SERD), in patients with ER+/HER2- advanced or metastatic breast cancer: phase 1/2 study results. Breast Cancer Res. 2025;27(1):119.
14. Harding JJ, Jungels C, Machiels JP, Smith DC, Walker C, Ji T, et al. First-in-Human Study of INCB062079, a Fibroblast Growth Factor Receptor 4 Inhibitor, in Patients with Advanced Solid Tumors. Target Oncol. 2023;18(2):181-93.
15. Hilton J, Cristea M, Postel-Vinay S, Baldini C, Voskoboynik M, Edenfield W, et al. BMS-986158, a Small Molecule Inhibitor of the Bromodomain and Extraterminal Domain Proteins, in Patients with Selected Advanced Solid Tumors: Results from a Phase 1/2a Trial. Cancers (Basel). 2022;14(17).
16. Janku F, Kim TM, Iyer G, Spreafico A, Elez E, de Jonge M, et al. First-in-human study of naporafenib (LXH254) with or without spartalizumab in adult patients with advanced solid tumors harboring MAPK signaling pathway alterations. Eur J Cancer. 2024;196:113458.

17. Kang Z, Li S, Lin Y, Li Y, Mao Y, Zhang J, et al. A phase I dose-escalation study of SYHA1813, a VEGFR and CSF1R inhibitor, in patients with recurrent High-Grade Gliomas or Advanced Solid Tumors. Invest New Drugs. 2023;41(2):296-305.
18. Kristeleit R, Plummer R, Jones R, Carter L, Blagden S, Sarker D, et al. A Phase 1/2 trial of SRA737 (a Chk1 inhibitor) administered orally in patients with advanced cancer. Br J Cancer. 2023;129(1):38-45.
19. Kim SB, Bae KS, Lee JL, Lee WS, Ock CY, Lee MJ, et al. First-In-Human Dose Finding Study of Venadaparib (IDX-1197), a Potent and Selective PARP Inhibitor, in Patients With Advanced Solid Tumors. Cancer Med. 2025;14(4):e70576.
20. Li W, Wang Y, Xiong A, Gao G, Song Z, Zhang Y, et al. First-in-human, phase 1 dose-escalation and dose-expansion study of a RET inhibitor SY-5007 in patients with advanced RET-altered solid tumors. Signal Transduct Target Ther. 2024;9(1):300.
21. Ma Y, Zhao H, Xue J, Liu L, Yang N, Zhang Y, et al. First-in-human phase I study of TQ-B3139 (CT-711) in advanced non-small cell lung cancer patients with ALK and ROS1 rearrangements. Eur J Cancer. 2022;173:238-49.
22. Piperno-Neumann S, Carlino MS, Boni V, Loirat D, Speetjens FM, Park JJ, et al. A phase I trial of LXS196, a protein kinase C (PKC) inhibitor, for metastatic uveal melanoma. Br J Cancer. 2023;128(6):1040-51.
23. Pruis MA, Krebs MG, Plummer R, De Vos F, Angevin E, Prenen H, et al. A Phase I Trial of the Dual MET Kinase/OCT-2 Inhibitor OMO-1 in Metastatic Solid Malignancies Including MET Exon 14 Mutated Lung Cancer. Oncologist. 2023;28(12):e1248-e58.
24. Ruan DY, Huang WW, Li Y, Zhao Y, Shi Y, Jia Y, et al. Safety, pharmacokinetics and efficacy of HA121-28 in patients with advanced solid tumors and RET fusion-positive non-small-cell lung cancer: a multicenter, open-label, single-arm phase 1/2 trial. Signal Transduct Target Ther. 2025;10(1):62.
25. Schreiber AR, Kagihara JA, Corr BR, Davis SL, Lieu C, Kim SS, et al. First-in-Human Dose-Escalation Study of the Novel Oral Depsipeptide Class I-Targeting HDAC Inhibitor Bocodepsin (OKI-179) in Patients with Advanced Solid Tumors. Cancers (Basel). 2023;16(1).
26. Shi Y, Hu X, Li X, Gong C, Wang K, Li Y, et al. Ficonalkib (SY-3505) in Advanced ALK-Positive NSCLC: A Multicenter, Open-Label, Single-Arm, Phase 1/2 Study. J Thorac Oncol. 2024;19(6):898-911.
27. Subbiah V, Iannotti NO, Gutierrez M, Smith DC, Féliz L, Lihou CF, et al. FIGHT-101, a first-in-human study of potent and selective FGFR 1-3 inhibitor pemigatinib in pan-cancer patients with FGF/FGFR alterations and advanced malignancies. Ann Oncol. 2022;33(5):522-33.
28. Tsimberidou AM, Vining DJ, Arora SP, de Achaval S, Larson J, Kauh J, et al. Phase I Trial of TTI-101, a First-in-Class Oral Inhibitor of STAT3, in Patients with Advanced Solid Tumors. Clin Cancer Res. 2025;31(6):965-74.
29. Wang X, Luo Z, Chen J, Chen Y, Ji D, Fan L, et al. First-in-human phase I dose-escalation and dose-expansion trial of the selective MEK inhibitor HL-085 in patients with advanced melanoma harboring NRAS mutations. BMC Med. 2023;21(1):2.
30. Yap TA, Fontana E, Lee EK, Spigel DR, Højgaard M, Lheureux S, et al. Camonsertib in DNA damage response-deficient advanced solid tumors: phase 1 trial results. Nat Med. 2023;29(6):1400-11.
31. Yap TA, Tolcher AW, Plummer R, Mukker JK, Enderlin M, Hicking C, et al. First-in-Human Study of the Ataxia Telangiectasia and Rad3-Related (ATR) Inhibitor Tuvusertib (M1774) as Monotherapy in Patients with Solid Tumors. Clin Cancer Res. 2024;30(10):2057-67.
32. Yap TA, Choudhury AD, Hamilton E, Rosen LS, Stratton KL, Gordon MS, et al. PF-06952229, a selective TGF-β-R1 inhibitor: preclinical development and a first-in-human, phase I, dose-escalation study in advanced solid tumors. ESMO Open. 2024;9(9):103653.
33. Zhang J, McAndrew NP, Wang X, Du Y, DiCarlo B, Wang M, et al. Preclinical and clinical activity of DZD1516, a full blood-brain barrier-penetrant, highly selective HER2 inhibitor. Breast Cancer Res. 2023;25(1):81.

34. Zhao S, Zhou H, Yang N, Wang Z, Jin W, Ma Y, et al. Safety, Efficacy, and Biomarker Analysis of Deulorlatinib (TGRX-326) in Anaplastic Lymphoma Kinase-Positive NSCLC: A Multicenter, Open-Label, Phase 1/1b Trial. J Thorac Oncol. 2025;20(6):750-62.
